# Full-Length Transcriptome and Gene Expression Analysis of Different *Ovis aries* Adipose Tissues Reveals Transcript Variants Involved in Lipid Biosynthesis

**DOI:** 10.3390/ani14010007

**Published:** 2023-12-19

**Authors:** Lixia An, Yangyang Pan, Mengjiao Yuan, Zhonghao Wen, Liying Qiao, Weiwei Wang, Jianhua Liu, Baojun Li, Wenzhong Liu

**Affiliations:** 1 College of Animal Science, Shanxi Agricultural University, Jinzhong 030801, China; 13934410131@139.com (L.A.); pyybiology@163.com (Y.P.);; 2 School of Food & Environment, Jinzhong College of Information, Jinzhong 030801, China

**Keywords:** adipose tissue, full-length transcriptome, new transcript, RNA sequencing, sheep

## Abstract

**Simple Summary:**

The substantial accumulation of fat in the bodies of sheep will reduce the lean meat percentage to a certain extent, affect the carcass quality, and reduce the production efficiency. Therefore, there is a growing focus on investigating variations in adipose tissue formation across different regions, deciphering genetic regulatory mechanisms, reducing unnecessary fat hoarding, and assessing the utility of fat in current research. In this study, the expression of subcutaneous, tail, and visceral fat transcripts of Dorper × Hu sheep was quantified at the transcriptome level by combining the second- and third-generation sequencing technologies. The results revealed 185 (3198), 394 (3592), and 83 (3286) differentially expressed genes (transcripts) between tail and subcutaneous, tail and visceral, and subcutaneous and visceral adipose tissues, respectively. The results revealed that certain differentially expressed genes and transcripts were related to fat metabolism. These results provide a comprehensive dataset for further verification of the regulatory pathway linked to fat metabolism in sheep.

**Abstract:**

Sheep have historically been bred globally as a vital food source. To explore the transcriptome of adipose tissue and investigate key genes regulating adipose metabolism in sheep, adipose tissue samples were obtained from F1 Dorper × Hu sheep. High-throughput sequencing libraries for second- and third-generation sequencing were constructed using extracted total RNA. Functional annotation of differentially expressed genes and isoforms facilitated the identification of key regulatory genes and isoforms associated with sheep fat metabolism. SMRT-seq generated 919,259 high-accuracy cDNA sequences after filtering. Full-length sequences were corrected using RNA-seq sequences, and 699,680 high-quality full-length non-chimeric (FLNC) reads were obtained. Upon evaluating the ratio of total lengths based on FLNC sequencing, it was determined that 36,909 out of 56,316 multiple-exon isoforms met the criteria for full-length status. This indicates the identification of 330,375 full-length FLNC transcripts among the 370,114 multiple-exon FLNC transcripts. By comparing the reference genomes, 60,276 loci and 111,302 isoforms were identified. In addition, 43,423 new genes and 44,563 new isoforms were identified. The results identified 185 (3198), 394 (3592), and 83 (3286) differentially expressed genes (transcripts) between tail and subcutaneous, tail and visceral, and subcutaneous and visceral adipose tissues, respectively. Functional annotation and pathway analysis revealed the following observations. (1) Among the differentially expressed genes (DEGs) of TF and SF tissues, the downregulation of *ACADL*, *ACSL6*, and *NC_056060.1.2536* was observed in SF, while *FFAR4* exhibited upregulation. (2) Among the DEGs of TF and VF tissues, expressions of *ACADL*, *ACSL6*, *COL1A1*, *COL1A2*, and *SCD* were downregulated in VF, with upregulation of *FFAR4*. (3) Among SF and VF expressions of *COL1A1*, *COL1A2,* and *NC_056060.1.2536* were downregulated in VF. Specific differentially expressed genes (*ACADL*, *ACSL6*, *COL1A1*, *COL1A2*, *FFAR4*, *NC_056060.1.2536*, and *SCD*) and transcripts (NC_056066.1.1866.16 and NC_056066.1.1866.22) were identified as relevant to fat metabolism. These results provide a dataset for further verification of the regulatory pathway associated with fat metabolism in sheep.

## 1. Introduction

As living standards improve, an increased emphasis on diet and health has elevated the demand for diverse and high-quality meat. Mutton, renowned for its richness in nutrients and essential trace elements, as well as its distinctive flavor and delectable meat, enjoys widespread popularity among consumers [1]. In 2021, there were approximately 1.23 billion sheep worldwide, contributing to a mutton production of approximately 9.96 million tons. In 2022, China’s mutton production reached 5.2453 million tons (https://data.stats.gov.cn/index.htm, accessed on 23 September 2023). Consequently, the sheep meat industry plays a crucial role in the advancement of livestock husbandry in China.

The Hu sheep, a distinctive local breed in China, stands out for its notable characteristics. Ewes of this breed are characterized by docility, year-round estrus, and a remarkable reproductive capacity, yielding two lambs annually or three lambs biannually on average, with an average lambing rate exceeding 200% [2]. Additionally, these ewes exhibit high adaptability and high reproduction rates. Consequently, they are the primary maternal choice for the industrial sheep meat production in China [3,4]. On the other hand, Dorper sheep, originating from South Africa and resulting from a cross between the South African Persian Blackhead regional breed and the English Dorset Horn, boasts strengths such as adaptability, resistance to disease, early growth and development, excellent carcass quality, and high lean meat percentage [5]. Introduced to China in 2001 [6], Dorper sheep have since proliferated across provinces, contributing to the diversification of the species. In pursuit to breeding new sheep lines characterized by high fertility, favorable carcass traits, and high lean meat percentage, the crossbreeding model of a maternal Hu sheep and a paternal Dorper sheep has been widely used in China. Hence, evaluating fat metabolism in the Dorper × Hu *F1* generation at the transcriptome level could provide a scientific basis for the genetic improvement of sheep meat and its economic traits.

A transcript represents mature mRNA resulting from gene transcription, capable of encoding one or more proteins. Gene transcripts undergo diverse splicing to generate various isoforms. With the commercialization of sequencing platforms and the reduced sequencing costs, transcriptome sequencing technology has found widespread application in animal transcriptome studies, including pigs [7], chickens [8], beef cattle [9], goats [10], and sheep [11]. Previous studies on sheep fat metabolism have primarily focused on short-read RNA-seq data from second-generation sequencing platforms [11,12,13]. The primary objectives of these studies were to describe gene expression levels and explore the differentially expressed genes related to lipid metabolism. However, RNA-seq has inherent drawbacks. (1) Fragments must be broken and then spliced, yielding incomplete fragments; (2) resultant transcripts are of lower quality, potentially leading to inaccurate annotation; (3) read lengths are limited and may not span the entire transcript. In contrast, full-length PacBio SMRT Iso-seq offers several advantages: (1) direct acquisition of complete transcripts without the need for fragment breaking and splicing; (2) longer reads, providing more comprehensive transcriptome information; (3) applicability to the analysis of complex transcriptome structures [14]. Thus, Iso-seq serves to address the limitations of RNA-seq. Iso-seq has been successfully employed for transcriptome analysis in various organisms, including beef cattle [15], crayfish [16], insects [17], and plants [14]. However, limited studies [3] have utilized PacBio SMRT Iso-seq for investigating isoforms associated with fat metabolism in sheep.

Our objective was to advance understanding of the sheep adipose tissue transcriptome by integrating RNA-seq and Iso-seq. We employed a comprehensive approach involving differential expression analysis and annotation to identify genes and transcripts implicated in fat metabolism. To this end, subcutaneous, tail, and visceral adipose tissues of sheep were collected, and full-length transcripts were obtained without splicing by utilizing the ultra-long read length of third-generation sequencing. In combination with ultra-high-throughput second-generation sequencing, transcript expression was quantified. Through functional annotation of differentially expressed genes (DEGs) and isoforms (DEIs), elements related to the regulation of lipid metabolism were characterized. Collectively, this study provides a theoretical foundation for studying the genetic regulation of fat deposition at different sites, as well as a dataset for exploring the sheep adipose tissue transcriptome.

## 2. Materials and Methods

### 2.1. Sample Collection

Four male Dorper × Hu crossbred sheep were raised to 8 months with standard nutrient formula. The sheep were euthanized using the bloodletting after PCB method, and subcutaneous fat (SF), visceral fat (VF), and tail fat (TF) were collected and snap frozen in liquid nitrogen or immobilized using 4% paraformaldehyde in phosphate-buffered saline. None of the sheep we used as experimental animals had been treated with tail amputation, and the fat tissue in the middle of the left half of the tail of each sheep was taken as the tail fat sample for this study.

### 2.2. Sequencing Library Preparation

**Figure 1:**
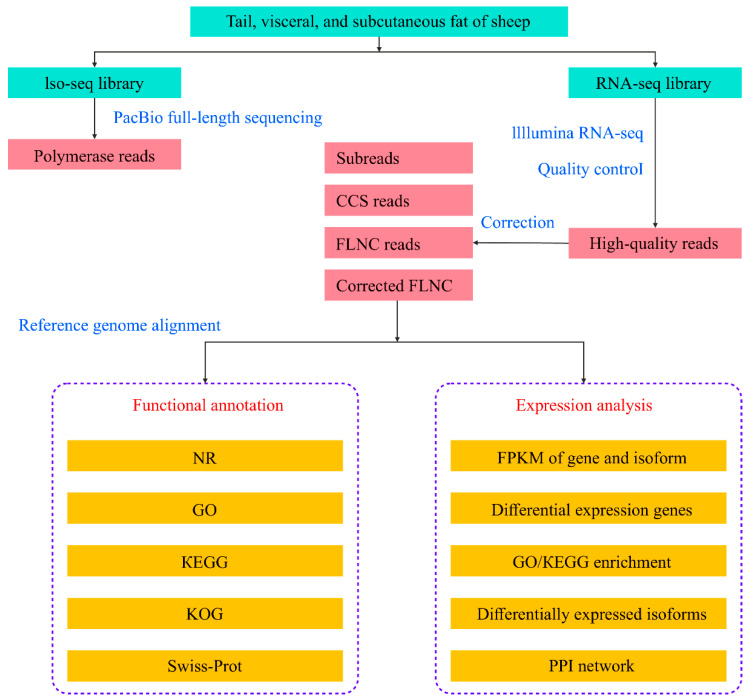
Hybrid sequencing workflow. Library preparation (blue highlighted text), pretreatment of raw sequencing reads (pink highlighted text), and functional analysis (orange highlighted text). CCS, circular consensus sequences; FLNC, full-length non-chimeric; NR, Non-Redundant Protein Sequence Database; GO, Gene Ontology; KEGG, Kyoto Encyclopedia of Genes and Genomes; KOG, Eukaryotic Orthologous Groups; FPKM, fragments per kilobase million; PPI, protein–protein interaction.

Sequencing and data analysis procedures are shown in Figure 1. Library preparation and sequencing followed previously reported methods with slight modifications [10]. Total RNA was extracted from frozen adipose tissues using TRIzol (Takara, Dalian, China) [12]. RNA concentration and integrity were validated using a Nanodrop™ 2000 Spectrophotometer (Thermo Fisher Scientific, Waltham, MA, USA). The integrity of 1 μg of RNA at a concentration of 200 ng/μL with an OD_260/280_ ratio of 1.8–2.2 was verified using an Agilent 2100 Bioanalyzer (Agilent Technologies, Santa Clara, CA, USA). Contaminating genomic DNA was excluded through electrophoresis. Qualified RNA was used for full- and short-length cDNA synthesis. To obtain a full-length sequencing library, RNA from all 12 adipose tissue samples was mixed equally by weight. A Clontech SMARTer PCR cDNA Synthesis Kit (Takara Bio, Kusatsu, Japan) was used to synthesize the cDNA library. Full-length cDNA was purified using AMPure PB Beads (Beckman Coulter, Brea, CA, USA).

To prepare the short-length sequencing libraries, mRNA was enriched using magnetic beads with Oligo (dT), followed by a fragmentation step. First-chain cDNA was synthesized using the fragmented mRNAs as templates. After second-chain cDNA synthesis, cDNA was purified using a gel extraction kit and then underwent sticky-end repair and polyadenylation at the 3′-end. Libraries were amplified using PCR to obtain sufficient cDNA for short-length sequencing. All libraries used for short- and full-length sequencing were quantified using an Agilent 2100 Bioanalyzer and Qubit 3.0 Fluorometer (Thermo Fisher Scientific). Short-length cDNA libraries were sequenced using an Illumina PE150 (Illumina, San Diego, CA, USA), while the full-length cDNA library was sequenced by Frasergen, Wuhan, China, using a PacBio Sequel II (Pacific Biosciences, Menlo Park, CA, USA).

### 2.3. Initial Analysis of Full-Length Sequencing Data

Raw data from the PacBio Sequel II were pre-treated using the SMART Link v10.1 platform with the following parameters: minimum subread length = 50, maximum subread length = 15,000, minimum number of passes = 3, and minimum predicted accuracy = 0.99. Circular consensus sequences (CCSs) were formed from subreads split from the same polymerase reads after self-correction. Concatemers of 5′- and 3′-end primers of CCS were recognized and, thus, full-length non-chimeric (FLNC) reads were identified. FLNC reads with polyadenylate (poly(A)) tails were used for subsequent analysis. Sequencing errors were corrected through a minimum of three passes of molecules through a zero-mode waveguide in the sequencing cell. Additionally, as short-length sequencing has high accuracy, the full-length sequences were further corrected by short-length reads using LoRDEC 0.9 (parameters: −k 21 −s 3) [18]. Full-length sequences before and after correction were mapped to the sheep reference genome using GMAP (parameters: no chimeras, n 10 min, and intron length = 10 K 800,000) [19], together with a percentage of identity (PID) calculation. Sequences with a high PID were retained for further analysis.

To identify gene loci and isoforms, transcripts were aligned against the sheep reference genome (http://www.ncbi.nlm.nih.gov/assembly/GCF_016772045.1/, accessed on 10 March 2022). Transcripts with the same mapping directions, >20% initial overlapping sequences, and >20% overlapping sequences in at least one exon were considered to have originated from the same locus and were, thereafter, used to identify isoforms. Redundant and false-positive transcripts were filtered from the transcripts originating from the same locus. Transcripts that had the same splicing sites as others despite being shorter and those that had the same sequences but were deficient in 5′-UTRs were considered redundant. Positive transcripts covered at least two FLNC reads or the junction of a single FLNC read annotated by the reference genome or our short-length sequencing. Otherwise, the transcripts were considered false positives. Because there were fragmentary non-full-length transcripts in the sequencing reads due to errors or RNA degradation during library preparation, the ratio of full-length transcripts to total transcripts was evaluated. FLNC transcripts and isoforms identified in this study covering the splicing donor sites of annotated isoforms in the reference genome were considered full-length. Their ratios to total FLNC transcripts and isoforms indicated the quality of the library preparation and sequencing.

### 2.4. Identification and Annotation of Novel Genes and Isoforms

The loci and isoforms identified in this study were mapped to the annotated genome, and their similarities were compared. Loci with <20% overlap with the annotated genome, or >20% overlap but in the opposite orientation, were considered as novel genes. Isoforms that had one or more new splicing sites when compared with the annotated genome were considered as novel isoforms. Novel genes and isoforms were mapped to the Non-Redundant Protein Sequence Database (NR), the Gene Ontology (GO) resource [20], the Eukaryotic Orthologous Groups (KOG) [21], the Kyoto Encyclopedia of Genes and Genomes (KEGG) [22], and Swiss-Prot [23] to obtain the amino acid sequences that had the highest similarities with annotations and were, thus, annotated with functional information. Additionally, the coding potential of novel isoforms with no hits in NR, KOG, KEGG ontology (KO), or Swiss-Prot was evaluated using the Coding Potential Assessing Tool (CPAT) 1.2.4 [24].

### 2.5. Recognition of Alternative Splicing, Fusion Genes, and Alternative Polyadenylation

With the help of the long reads produced by third-generation sequencing, transcript diversity was more directly and accurately measured. Astalavista [25] was used to classify the alternative splicing (AS) events of isoforms as exon skipping (ES), alternative 5′ donor sites (AD), alternative 3′ acceptor sites (AA), and intron retention (IR). Fusion genes from FLNC transcripts containing 5′ and 3′ partner genes were identified by fusion gene detection software (Frasergen, Wuhan, China) developed by Frasergen. Fusion genes were classified as inter- or intrachromosomal according to partner genes on the same or different chromosomes, respectively. Alternative polyadenylation (APA) was recognized based on the aligned location of the 5′-FLNC sequence on the genome. The number of poly-A sites in each gene and the number of corresponding transcripts were analyzed using Tapis [26]. The existence of APA was ensured by at least two FLNC transcripts.

### 2.6. Analysis of Short-Length Sequencing Data

Sequencing adaptors, low-quality reads, and contaminated reads were removed from the raw Illumina short-length sequencing (RNA-seq) data. The number of clean reads, data quantity, sequencing errors, Q20, Q30, and GC content were calculated. After quality control, clean reads were mapped to the sheep reference genome using Hisat2 [27]. We subsequently combined the annotated transcripts from the database with transcripts obtained from our PacBio sequencing to form a new transcript library. Reads from RNA-seq were mapped to the new library using Bowtie2 [28]. After mapping, fragments per kilobase million bases (FPKM) of each gene and transcript were calculated using RSEM [29] based on the number of reads mapped to each transcript. Commonly expressed genes and isoforms were visualized using a Venn diagram. Correlations among genes from different samples were calculated to indicate sample similarity.

DEGs and DEIs were identified using DEseq2 [30]. Briefly, the read count of each gene and isoform was normalized to calculate a *p*-value. Multiple hypothesis testing and correction were performed to obtain a false discovery rate (FDR) value. Those with |log2(fold-change)| > 1 and adjusted *p* < 0.05 were considered DEGs and DEIs. Among the DEGs, transcription factors were identified according to the AnimalTFDB [31] and PlnTFDB databases using HMMER [32]. MA and volcano plots were used to show the distribution of the FDR and fold-change, while DEGs and DEIs were clustered to show their expression patterns in different samples. Enriched functions of DEGs and DEIs were explored using the OmicShare tools (www.omicshare.com/tools, accessed on 23 March 2022) in GO and KEGG database. GO terms and KEGG pathways that had an FDR ≤ 0.05 were considered enriched. The STRING protein interaction database [33] was used to construct a protein–protein interaction (PPI) network of DEGs and DEIs followed by visualization using Cytoscape 3.9.0 [34].

### 2.7. Validation of Sequencing Accuracy

Quantitative real-time PCR (qRT-PCR) was performed to validate the accuracy of RNA-seq. Primers were designed to cross the exon–exon junctions of transcripts (Table S1). Briefly, 1 μg of RNA was reverse transcribed using a PrimeScript™ RT Reagent Kit with gDNA Eraser (Takara Bio). TB Green^®^ Premix Ex Taq™ II was used to measure the mRNA abundance in a CFX Connect Real-Time PCR Detection System (Bio-Rad, Hercules, CA, USA). The relative expression of mRNA was normalized to that of *B2M* using the 2^−ΔΔCT^ method. Data are presented as mean ± SEM (*n* ≥ 3) and were visualized using GraphPad Prism 8 (GraphPad Software, San Diego, CA, USA).

## 3. Results

### 3.1. Overall Description of Full-Length Sequencing and Error Correction

**Figure 2:**
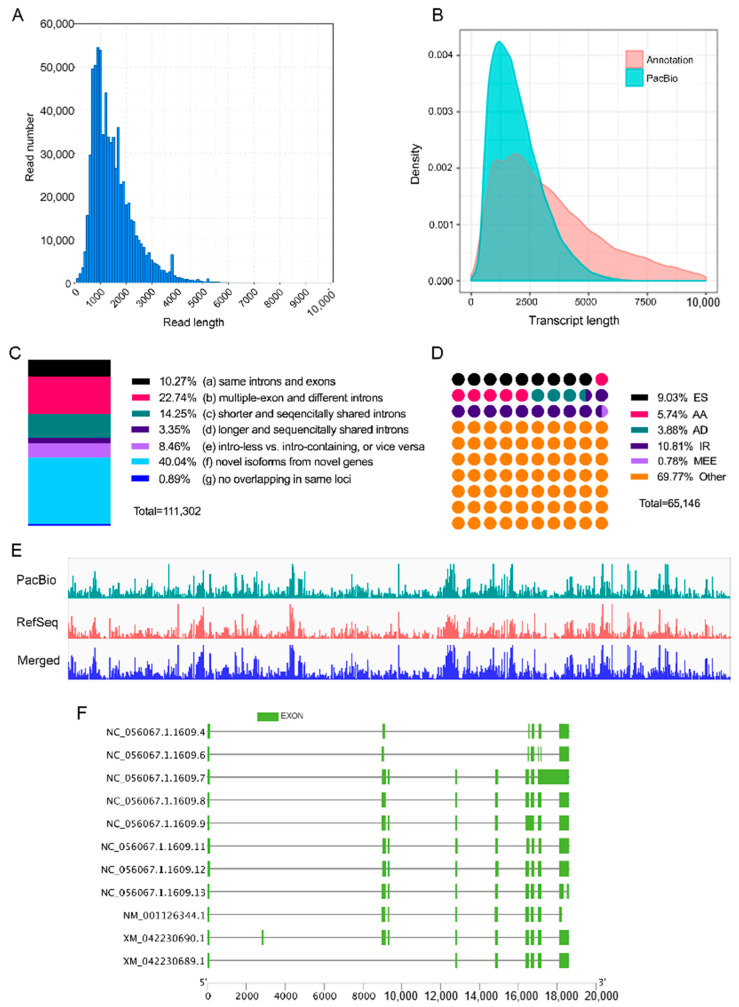
Full-length sequencing annotation of the sheep transcriptome. (**A**) Length distribution of full-length non-chimeric (FLNC) reads; (**B**) length distribution of isoforms based on NCBI RefSeq annotation and full-length PacBio sequencing; (**C**) structural categories of isoforms identified by full-length sequencing; (**D**) numbers of alternative splicing events. AA, alternative acceptor site; AD, alternative donor site; ES, exon skipping; IR, intron retention; MEE, mutually exclusive exon. (**E**) Integrative Genomic Viewer (IGV) track of transcript annotation from the sheep reference genome (RefSeq), long-read isoform sequencing (PacBio), and the improved RefSeq using PacBio (merged); (**F**) genomic structure of BCKDHA isoforms. The isoforms headed by NC_ are the novel isoforms identified in this study. The isoforms headed by NM_ and XM_ are the annotated isoforms using the NCBI database.

RNA extracted from the VF, TF, and SF of all four sheep was pooled for PacBio full-length transcriptome sequencing. Given the circular nature of sequences produced by this platform, the resulting high-quality reads were termed polymerase reads. A total of approximately 75.6 Gbp of data was obtained, representing 1,357,451 polymerase reads. The lengths of the polymerase reads varied, with a maximum length of 395,143 bp, a minimum length of 51 bp, and an average length of 55,691 bp (Figure S1A). After the removal of sequencing adaptors, 52,410,331 subreads were obtained, with an average length of 1362 bp (Figure S1B). Circular Consensus Sequences (CCSs) were generated following correction using the sequences that passed through the same zero-mode waveguide, as described in the “Initial Analysis of Full-Length Sequencing Data” section. In total, 919,259 CCS were identified, with an average length of 12,653 bp (Figure S1C). The quality and length distributions of these CSSs are illustrated in Figure S1D and Figure S1E, respectively. Further examination of the 5′ primer, 3′ primer, poly(A) tail, and their spatial relationships on the CCS revealed that 743,169 CCS contained both 5′ and 3′ primers classifying them as full-length reads. Among these, 720,512 were classified as full-length non-chimeric (FLNC) reads, and 720,248 of these FLNC reads possessed poly(A) tails. For further analysis, FLNC reads with poly(A) tails were utilized, showing an average length of 1452 bp, with a maximum length of 7023 bp (Figure 2A).

Owing to the high accuracy of second-generation sequencing, the full-length reads obtained from third-generation sequencing were corrected using the short reads. The mean PID value of the total reads after correction was 99.03%, which was higher than that before (98.97%; Table 1). Approximately 33.49% of FLNC reads had a higher PID value after correction.

Sequences with a high PID value before or after correction were merged into a new FLNC library (merged). All FLNC transcripts were mapped to the sheep reference genome (http://www.ncbi.nlm.nih.gov/assembly/GCF_016772045.1/, accessed on 10 March 2022); the results showed that the merged library had more high-quality mapped reads than did the pre- or post-correction sequences (97.14%), along with a lower poor-PID ratio (2.02%; Table 2).

### 3.2. Loci and Isoform Identification in Full-Length Sequencing

Following sequence correction and genome mapping, known loci and isoforms were identified from the merged FLNC library. A total of 111,302 isoforms were annotated from the high-PID sequences, surpassing the 68,644 isoforms present in the reference genome (Table 3). Full-length sequencing revealed the identification of more 1000–3000 bp loci compared to the reference annotation (Figure 2B and Table S2). To assess the integrity of the identified isoforms, the ratio of full-length isoforms was evaluated. Multiple-exon isoforms sharing splicing donor sites and directions with those in the reference genome were considered full-length isoforms. In summary, 36,909 out of 56,316 multiple-exon isoforms were deemed full-length (65.54%), representing 330,375 full-length FLNC transcripts in 370,114 multiple-exon FLNC transcripts (89.26%).

Novel loci and isoforms were identified through comprehensive full-length sequencing. A total of 66,739 isoforms, encompassing both known and novel variants, were identified. While 15,235 known loci eluded identification, 55,301 of their corresponding isoforms exhibited expression in our samples. Conversely, 44,563 isoforms resisted mapping to the reference genome, yet our algorithm successfully mapped 43,423 novel loci. Upon functional annotation of these novel isoforms using diverse databases, 65.81% displayed one or more records (Table S3). KEGG analysis highlighted 61 novel isoforms associated with lipid metabolism, with several others enriched in energy metabolism pathways (Figure S2A). GO analysis unveiled 6308 and 1906 novel isoforms with potential involvement in metabolic and developmental processes, respectively (Figure S2B).

### 3.3. Transcriptome Diversity

Long-read sequencing delineated a comprehensive transcriptome, categorizing isoform structures into seven categories (Figure 2C). Among these, multiple-exon isoforms (11,438) shared identical splicing sites with reference isoforms, constituting 10.27% of the total PacBio isoforms (category A). Approximately half of the PacBio isoforms exhibited diverse exon and intron arrangements compared to the reference isoforms, falling into categories B, C, D, and E. A notable proportion (40.04%) of PacBio and reference isoforms originated from different genes, either in the same or reverse directions (category F). A minority of PacBio isoforms (0.89%) emanated from the same loci as the reference isoforms but lacked overlapping sequences (category G).

Full-length isoforms served as the foundation for identifying alternative splicing (AS) events, resulting in the characterization of 65,146 events classified into six types. Among these, 9.03% were exon skipping (ES), 5.74% alternative acceptor sites (AA), 3.88% alternative donor sites (AD), 10.81% intron retention (IR), and 0.78% mutually exclusive exons (MEE) (Figure 2D).

Fusion genes were predicted using full-length reads and chimeric genes characterized by both 5′ and 3′ partner genes within the same modulating sequences were identified as fusion genes. A total of ten fusion genes were discovered, with nine having partner genes originating from the same chromosome, and one exhibiting a partner gene from a different chromosome (Table S4).

The alternative polyadenylation (APA) of each mRNA precursor was validated by a minimum of two full-length non-chimeric (FLNC) transcripts. Overall, 5291 genes demonstrated APA, with 84 of them featuring more than five APA sites (Table S5). Notably, *BZW1*, *FABP4*, *LOC101110189*, *LPL*, *PNISR*, *SCD*, and *SPCS3* exhibited over 10 APA sites. The coding potential of the novel isoforms, lacking matches in the NR, KOG, KO, or Swiss-Prot database, was assessed using CPAT.

### 3.4. Overall Description of Short-Read Sequencing Data

Clean Illumina RNA-seq short reads were acquired following the removal of contaminants and adaptors from the raw reads. Each sequencing library generated an average of 22,566,819 clean reads, equivalent to approximately 6,770,045,800 pairs of clean bases (Table S6). Post-quality control, these clean reads were aligned to the sheep reference genome and the merged PacBio + RefSeq transcriptome library. Between 91.02% and 96.14% of the total reads of each sample were mapped to the reference genome. The ratio of uniquely mapped reads varied from 74.37% to 88.65% (Table S7). Conversely, when mapped to the merged transcriptome library, the ratio of total mapped reads ranged from 74.55% to 84.99% and the ratio of unique mapped reads was less than 10% (Table S8).

Expression levels of genes and isoforms were normalized to FPKM. An FPKM value >60 was considered highly expressed, representing approximately 3% of genes and 1.5% of isoforms in each library (Tables S9 and S10). Examining expressed genes and isoforms (FPKM > 0) within tissue subgroups, it was observed that 14,307, 16,491, and 14,675 genes, as well as 15,661, 21,784, and 17,559 isoforms, were commonly expressed in TF, VF, and SF, respectively.

### 3.5. DEGs and DEIs

**Figure 3:**
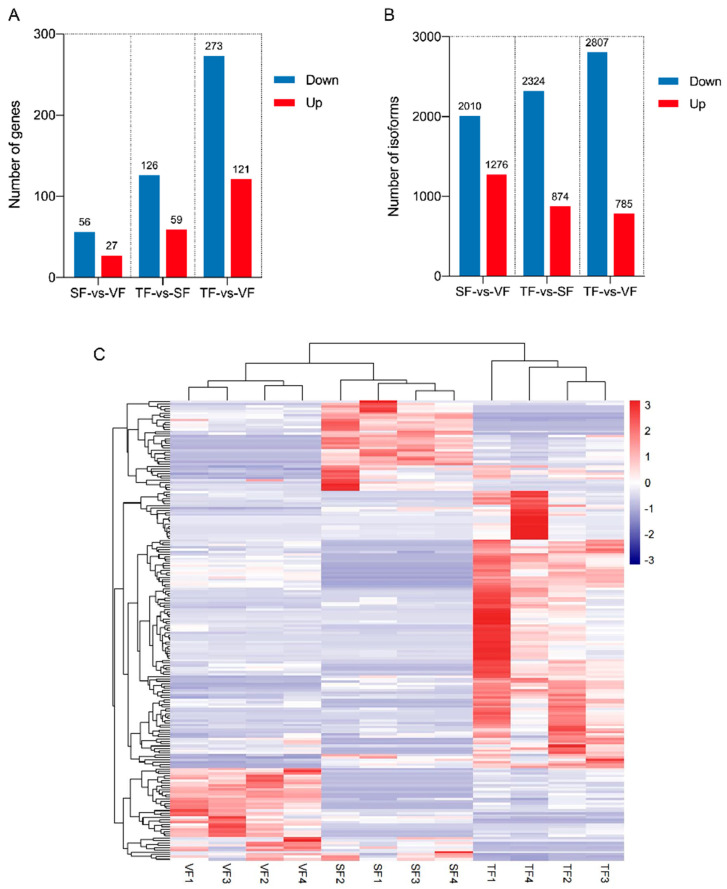
Short-read sequencing identified multiple differentially expressed genes and isoforms (DEGs and DEIs). (**A**) DEGs between different types of adipose tissue. SF, subcutaneous fat; TF, tail fat; VF, visceral fat. (**B**) DEIs between different types of adipose tissue. (**C**) Heatmap of the expression patterns of DEGs (*n* = 200 genes).

DEGs and DEIs were identified based on criteria of |log2(fold-change)| > 1 and adjusted *p* < 0.05 across various sample groups. In TF vs. SF, 185 DEGs and 3198 DEIs were observed; in TF vs. VF, 394 DEGs and 3592 DEIs were identified; and in SF vs. VF, 83 DEGs and 3286 DEIs were detected (Figure 3A,B). The expression patterns of the top DEGs in each sample are visually represented as a heatmap (Figure 3C).

### 3.6. Functional Annotation of DEGs and DEIs

**Figure 4:**
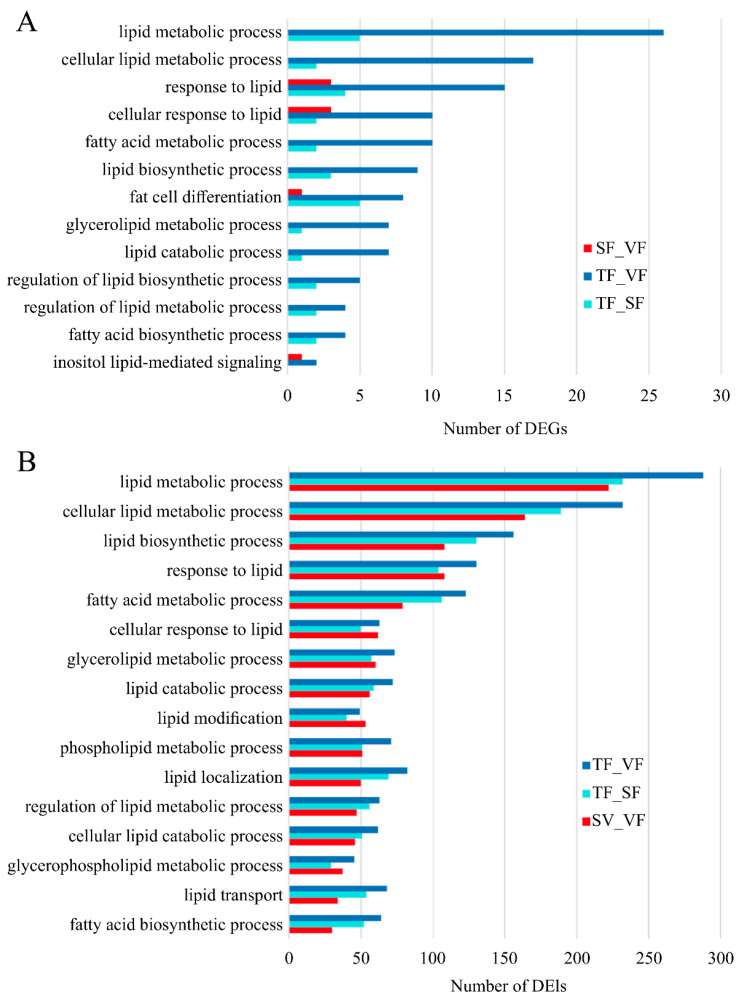
Top Gene Ontology biological processes of differentially expressed genes and isoforms among different groups. SF, subcutaneous fat; TF, tail fat; VF, visceral fat. (**A**) Top Gene Ontology biological processes of differentially expressed genes among dirrerent groups. (**B**) Top Gene Ontology biological processes of differentially expressed isoforms among dirrerent groups.

For functional annotation of DEGs and DEIs, GO and KEGG analyses were conducted. The most enriched biological process identified was lipid metabolism, linking DEGs (Figure 4A) and DEIs (Figure 4B) to adipose metabolism. Notably, SF and VF samples exhibited fewer DEGs associated with lipid metabolic processes, while multiple DEIs were implicated in potential roles. TF and VF samples displayed a high abundance of DEGs and DEIs closely related to lipid metabolism compared to other groups.

GO enrichment analysis of DEGs revealed the following. (1) Among the DEGs of TF and SF tissues, five genes (*FFAR4*, *INSIG1*, *NC_056075.1.194*, *NR4A1*, and *PTGS2*) were enriched in biological processes related to adipocyte differentiation, while three genes (*ALB*, *FFAR4*, and *NC_056075.1.194*) were associated with molecular functions related to fatty acid binding proteins. (2) Among the DEGs of TF and VF tissues, five genes (*ALB*, *FFAR4*, *NC-056075.1.294*, *S100A8*, and *S100A9*) showed highly significant enrichment (*p* < 0.01) in molecular functions related to fatty acid binding. Notably, the novel gene *NC-056075.1.294* was implicated in the biological process of fatty acid binding. Furthermore, *ACVR1, APOE*, and *MEST* were significantly enriched (*p* < 0.05) in the biological process of lipid storage, *ALOX15* and *SCD* in the biological process of unsaturated fatty acid biosynthesis, and *PHYH* in the molecular function of phytanoyl-CoA dioxygenase activity. *APOE* was also involved in diverse processes such as low-density lipoprotein particle receptor catabolism, its regulation, lipid transport across the blood–brain barrier, and positive regulation of lipid transport involved in lipid storage across the blood–brain barrier was related to lipid transport involved in lipid storage. Additionally, *ACLY*, *ALOX15*, *INSIG1*, and *SCD* were significantly enriched (*p* < 0.05) in fatty acid biosynthesis, while *S100A8* and *S100A9* may play a role in long-chain fatty acid binding. *DCN* was highly significantly enriched (*p* < 0.01) in the negative regulation of phosphorylation, and *FFAR4*, *INSIG1*, and *NC_056075.1.194* were highly significantly enriched (*p* < 0.01) in adipocyte differentiation.

KEGG analysis of DEGs revealed the following. (1) Among the DEGs of SF and VF tissues, five genes (*COL1A1*, *COL1A2*, *LOC101111528*, *LOC121819503*, and *NC_056060.1.2536*) were associated with lipid metabolism, with *LOC101111528* enriched in primary bile acid biosynthesis and the PPAR signaling pathway. The novel gene *NC_056060.1.2536* showed enrichment in the cell adhesion pathway, while *LOC121819503* was significantly enriched (*p* < 0.05) in bile, gastric acid, pancreatic, and salivary secretion. (2) Among the DEGs of TF and SF tissues, *ACADL* and *ACSL6* were significantly enriched (*p* < 0.05) in fatty acid metabolism, degradation, and the PPAR signaling pathway. *ACSL6* additionally exhibited enrichment in fatty acid biosynthesis and the adipocytokine signaling pathway, highlighting its crucial roles in fatty acid metabolism. *PTGS2* was enriched in arachidonic acid metabolism and the regulation of lipolysis in adipocytes, suggesting a potential involvement of arachidonic acid in lipid metabolism. Five novel genes, including *LOC101117229* and *LOC101122246* (significantly enriched (*p* < 0.05) in fat digestion and absorption and glycerolipid metabolism) and *NC_056070.1.1126*, *NC_056055.1.4467*, and *NC_056060.1.2536* (enriched in cell adhesion), were identified, establishing their close association with lipid metabolism. (3) Among the DEGs of TF and VF tissues, *ACSL5* and *ACSL6* were concurrently enriched in fatty acid metabolism, the PPAR signaling pathway, fatty acid biosynthesis and degradation, and the adipocytokine signaling pathway. *PPT1* was enriched in fatty acid metabolism and the fatty acid elongation pathway. *SCD* demonstrated enrichment in fatty acid metabolism, the PPAR signaling pathway, and unsaturated fatty acid biosynthesis. Additionally, *LOC101119706* and *LOC101112384* were simultaneously enriched in the steroid hormone biosynthesis pathway.

The GO enrichment analysis of DEIs revealed the following. (1) Within the DEIs identified in TF and VF tissues, the newly discovered transcripts NC_056067.1.450.7 and NC_056066.1.1866.16 were highly significantly enriched (*p* < 0.01) in the brown fat cell differentiation (BFCD) term. (2) Within the DEIs of SF and VF tissues, the new transcripts NC_056067.1.450.7 and NC_056066.1.1866.16 were highly significantly enriched (*p* < 0.01) in the white fat cell differentiation (WFCD) term. (3) Within the DEIs of TF and SF tissues, a total of twelve DEIs were significantly (*p* < 0.05) enriched in the BFCD term, four of which were known transcripts (NM_001308565.1, NM_001130154.1, XM_004011190.5, XM_042229260.1), and eight of which were novel transcripts (NC_056054.1.2653.81, NC_056057.1.1197.32, NC_056057.1.1197.36, NC_056054.1.2653.150, NC_056054.1.2653.98, NC_056054.1.2653.111, NC_056065.1.1701.8, NC_056057.1.1197.21).

KEGG analysis of DEIs revealed the subsequent findings. (1) Within the DEIs of SF and VF tissues, the upregulated expression of NC_056079.1.98.44, in addition to its enrichment in the fatty acid metabolic pathway, demonstrated noteworthy associations with adipokine signaling pathway, fatty acid biosynthesis, fatty acid metabolism, and the PPAR signaling pathway. NC_056069.1.1013.11 exhibited enrichment in glycerophospholipid metabolism, glycerolipid metabolism and sphingolipid metabolism, fat digestion and absorption, and ether lipid metabolism. NC_056057.1.378.11 displayed enrichment in ECM-receptor interaction, PPAR signaling pathway, fat digestion and absorption, and adipokine signaling pathway. Furthermore, four downregulated DEIs were concurrently enriched in two pathways, namely fatty acid synthesis and fatty acid metabolism. They were identified as NC_056079.1.98.13, NC_056064.1.1869.40, NC_056079.1.98.43, and NC_056064.1.1869.106, with NC_056079.1.98.43 additionally enriched in the PPAR signaling pathway and adipokine signaling pathway. (2) Turning to the TF and SF tissues, three newly identified differentially expressed transcripts, NC_056079.1.98.25, NC_056064.1.1869.65, and NC_056079.1.98.49, exhibited enrichment in both fatty acid synthesis and fatty acid metabolism pathways. NC_056079.1.98.25 and NC_056079.1.98.49 demonstrated simultaneous enrichment in the adipokine signaling pathway, fatty acid degradation, and PPAR signaling pathway. The downregulated transcript NM_001136491.1 participated in fatty acid prolongation, fatty acid degradation, and fatty acid metabolism, and showed enrichment in the unsaturated fatty acid biosynthesis pathway. (3) Among the DEIs of TF and VF tissues, three upregulated DEIs (NC_056079.1.98.49, NC_056079.1.98.44, NC_056079.1.98.25) were enriched in fatty acid synthesis, fatty acid metabolism, fat cytokine metabolism, fatty acid degradation, and PPAR signaling pathway. NC_056059.1.1408.38 was also enriched in fatty acid metabolism, unsaturated fatty acid synthesis, and fatty acid elongation pathways. NC_056066.1.1544.9 was enriched in the adipocyte metabolism and steroid hormone synthesis pathways. In the downregulation of DEIs, NC_056056.1.2815.36 significantly (*p* < 0.05) influenced glycerophospholipid metabolism, glycerolipid metabolism, fat digestion and absorption. NC_056057.1.378.88 and NC_056057.1.378.99 were significantly (*p* < 0.05) enriched in the PPAR signaling pathway, adipocytokine signaling pathway, fat digestion and absorption pathway. NM_001136490.1 exhibited a highly significant enrichment (*p* < 0.01) in the fatty acid prolongation pathway, unsaturated fatty acid biosynthesis, and glycerol metabolism pathway.

### 3.7. Interactions between DEGs

**Figure 5:**
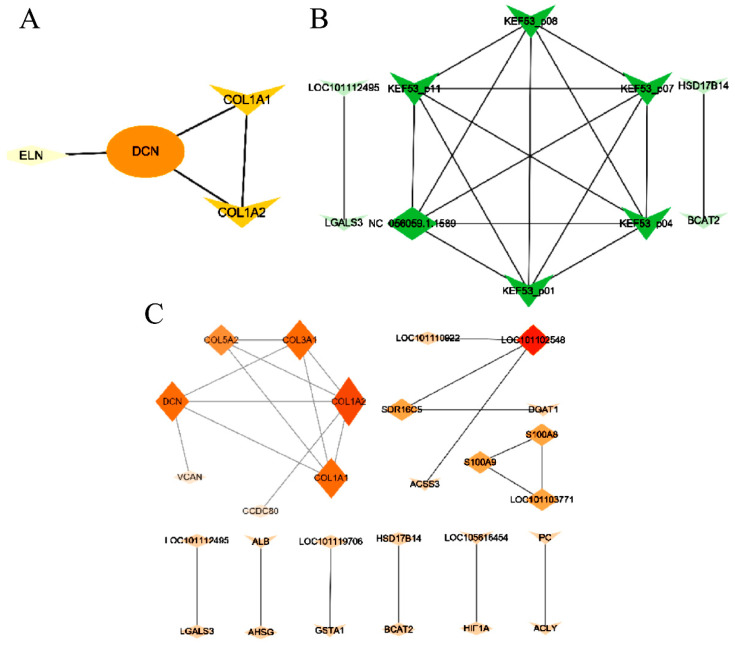
PPI network of DEGs among different tissues. PPI, protein–protein interaction; DEGs, differentially expressed genes. (**A**) PPI of DEGs among SF and VF; (**B**) PPI of DEGs among TF and VF; (**C**) PPI of DEGs among TF and SF. SF, subcutaneous fat; TF, tail fat; VF, visceral fat. The diamonds represent novel genes; the triangles represent known genes. Larger dots and darker colors indicate stronger interactions.

To explore the interactions between the DEGs among different groups, a PPI network was constructed using Cytoscape 3.9.0 (Figure 5). The midpoint represented a gene, and the edges represented direct interactions between genes. The larger the dot and the darker the color, the stronger the interaction in the PPI network. *DCN* interacted with *COL1A1*, *COL1A2*, and *ELN*, with mutual interaction between *COL1A1* and *COL1A2* in SF and VF tissues (Figure 5A). The PPI network of DEGs in TF and VF tissues shows that most DEGs interacted with downregulated *COL1A2*, followed by *COL3A1* and *COL1A1* (Figure 5B). In addition to the interactions between genes of the *COL* family, *DCN* also interacted with *COL3A1*, *COL1A2*, and *COL1A1*. *LOC101103771* interacted with *S100A8* and *S100A9* of the *S100A* family. By constructing the PPI network of DEGs in TF and SF tissues, 10 DEGs that interacted with one another were found (Figure 5C). Although the novel gene *NC_056059.1.1589* was not enriched in processes related to lipid metabolism, it interacted with *KEF53_p01*, *KEF53_04*, *KEF53_07*, *KEF53_08*, and *KEF53_09* in the *KEF53_p* family.

### 3.8. Interactions between DEIs

**Figure 6:**
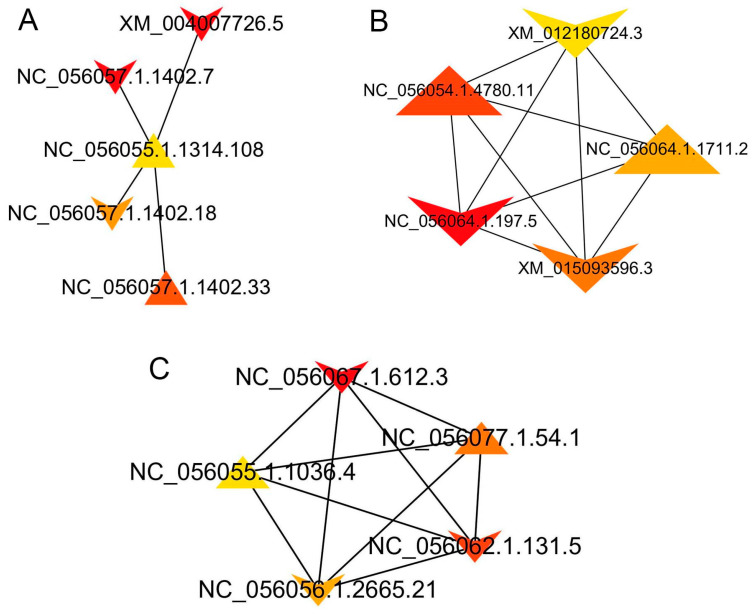
PPI network of DEIs among different tissues. PPI, protein–protein interaction; DEIs, differentially expressed isoforms. (**A**) PPI of DEIs among SF and VF; (**B**) PPI of DEIs among TF and SF; (**C**) PPI of DEIs among TF and VF. SF, subcutaneous fat; TF, tail fat; VF, visceral fat. The Vs represent downregulated DEIs; the triangles represent upregulated DEIs. Darker colors indicate higher MCC values.

To explore the interactions among differentially expressed isoforms (DEIs) related to lipid metabolism in different groups, a Protein Interaction (PPI) network was constructed using Cytoscape. The hub isoforms were identified based on the values of Matthews Correlation Coefficient (MCC) using the plugin CytoHubba (Figure 6). In the PPI network for SF and VF tissues, formed by 1651 nodes and 6365 edges, the top hub isoform NC_056055.1.1314.108 interacted with NC_056057.1.1402.7, NC_056057.1.14.02.18, NC_056057.1.1402.33, and XM_004007726.5 among the top five hub isoforms (Figure 6A). In the PPI networks for TF and SF tissues, formed by 1733 nodes and 8103 edges, the top five hub isoforms (NC_056064.1.197.5, NC_056054.1.4780.11, XM_012180724.3, NC_056064.1.1711.2, and XM_015093596.3) interacted with each other (Figure 6B). In the PPI network of DEIs in TF and VF tissues, the top five hub DEIs were NC_056067.1.612.3, NC_056077.1.54.1, NC_056062.1.131.5, NC_056056.1.2665.21, and NC_056055.1.1036.4, interacting with each other (Figure 6C).

The MCC values and the gene names of the top five hub DEIs in the PPI network among different tissues are listed in Table 4. The results showed that there were four DEIs correspinding to the gene *COL1A2* in the top five hub DEIs among SF and VF.

### 3.9. qRT-PCR Validation of DEGs

**Figure 7:**
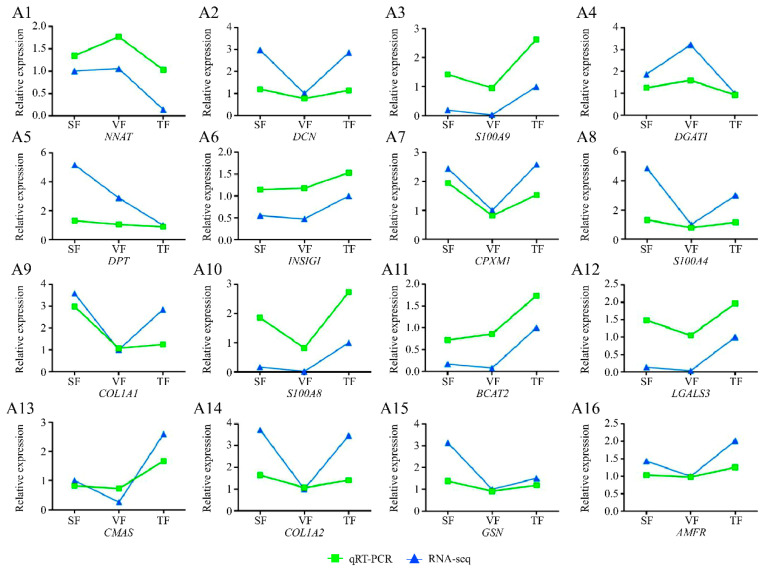
qRT-PCR validation of differentially expressed genes. (**A1**–**A16**) indicate the validation results of *NNAT*, *DCN*, *S100A9*, *DGAT1*, *DPT*, *INSIG1*, *CPXM1*, *S100A4*, *COL1A1*, *S100A8*, *BCAT2*, *LGALS3*, *CMAS*, *COL1A2*, *GSN*, and *AMFR*, respectively.

Sixteen DEGs were randomly selected for qRT-PCR validation (*AMFR*, *BCAT2*, *CMAS*, *COL1A1*, *COL1A2*, *CPXM1*, *DCN*, *DGAT1*, *DPT*, *GSN*, *INSIG1*, *LGALS3*, *NNAT*, *S100A4*, *S100A8*, and *S100A9*) (Figure 7). Their expression levels were simultaneously compared with the results of high-throughput sequencing. Of the sixteen DEGs in SF, VF, and TF, six (*COL1A1*, *COL1A2*, *CPXM1*, *DPT*, *GSN*, and *S100A4*) were highly expressed in SF, two (*DGAT1* and *NNAT*) in VF, and five (*BCAT2*, *INSIG1*, *LGALS3*, *S100A8*, and *S100A9*) in TF. Three (*DGAT1*, *DPT*, and *NNAT*) had their lowest expression levels in TF, and ten (*BCAT2*, *CMAS*, *COL1A1*, *COLA2*, *CPXM1*, *DCN*, *LGALS3*, *S100A4*, *S100A8*, and *S100A9*) in VF.

In conclusion, the qRT-PCR results showed a relatively consistent pattern with the RNA-seq data, suggesting that the findings from this study were partly reliable and could be employed for subsequent analyses, including variable shear, transcription factor, and lncRNA analysis.

## 4. Discussion

Adipose tissue is an important tissue regulating adipose development and lipid metabolism in livestock. Transcriptome sequencing was the preferrde technology to analyze gene expression and reveal biological characteristics. In this study, the transcriptome data of SF, TF, and VF tissues of Dorper × Hu hybrid F1 sheep were analyzed by combining second-generation RNA-seq and third-generation Iso-seq data.

### 4.1. Transcriptome Sequencing Data Quality

Jin M. et al. conducted a transcriptome analysis of six samples from Hu sheep and Tibet sheep, employing HISAT2 and Bowtie2 for reference genome comparison. The total comparison rates of clean reads were 86.03 and 65.41%, respectively, with unique comparison rates of 51.47 and 49.70%, respectively [13]. A study on adipose deposition in fat-tailed sheep reported that a unique comparison rate ranged from 70.42% to 76.36% [35]. In our study, after quality control, the unique comparison rate ranged from 74.37% to 88.65%. This indicates higher overall quality of the sequencing data, meeting the requirements for subsequent research. In a study using PacBio Iso-seq and Illumina RNA-seq to generate the transcriptome of three developmental stages of R. ferrugineus, 63,801 full-length transcripts were obtained with an average length of 2964 bp, surpassing that achieved by second-generation sequencing [14]. Yuan, Z. et al. combined pooled PacBio Iso-seq with six sheep RNA-seq datasets, obtaining 30,305,694 subreads, 360,901 CCSs, and 271,718 FLNCs after quality control [3]. In our work, a total of 52,410,331 subreads, 919,259 CCSs, and 720,512 FLNCs were obtained by PacBio iso-seq. After correcting high-quality reads via RNA-seq quality control, the total high-quality ratio reached 97.14%, surpassing previous reports [3,35]. In our study, 65,146 Alternative Splicing (AS) events with six patterns were identified, with 9.03% AS being Exon Skipping (ES), differing from some studies [3,14]. This discrepancy may arise from variations in detection methods and test animals. AS events contribute to transcript diversity leading to protein complexity and phenotype diversification [36]. Therefore, the identification of AS events is crucial for further exploring the mechanism of fat metabolism in different sheep tissues, and the full-length sequencing of PacBio Iso-seq provides technical support for this exploration.

### 4.2. Enrichment and Roles of DEGs in Fat Metabolism

Numerous molecular mechanisms associated with fat metabolism in sheep have been investigated [3,11,35]. However, the genetic mechanism at the transcriptional level of fat metabolism remains unclear. In this study, the analysis of Illumina RNA-seq data revealed the identification of 185, 394, and 83 DEGs between TF and SF, TF and VF, and SF and VF tissues, respectively.

In this study, *ACADL* and *ACSL6* were simultaneously enriched in fatty acid metabolism and degradation and the PPAR signaling pathway. *SCD* was enriched in fatty acid degradation, unsaturated fatty acid biosynthesis, and the PPAR signaling pathway. *COL1A1* and *COL1A2* were enriched in the extracellular matrix (ECM) receptor interaction pathway. Previous studies have indicated that long-chain acyl-CoA dehydrogenase could be used as a candidate gene to improve fat deposition traits [37]. *ACSL6* plays an important role in lipid synthesis, fatty acid catabolism, and biofilm remodeling [38]. *SCD1* is significantly correlated with fat deposition and fatty acid composition in skeletal muscle, displaying a negative correlation with saturated fatty acid content [39]. *COL1A1* and *COL1A2* are the *ECM* genes in adipose tissue, and their expression is directly associated with adipose tissue inflammation [40]. Furthermore, the novel gene identified in this study, *NC_056060.1.2536*, showed enrichment in the cell adhesion pathway, which is linked to atherosclerosis and plays a pivotal role in triglyceride metabolism [41]. Hence, *NC_056060.1.2536* is implicated in triglyceride metabolism.

*S100A8* and *S100A9,* members of the *S100* family, exhibit high expression of *S100A8* mRNA in mouse white adipose tissue (WAT) and differentiated 3T3-L1 adipose cells. The expression levels of *S100A8* mRNA in mature adipocytes of obese mice and interstitial vascular cells of lean mice are higher than those of lean mice [42]. This observation suggests a association between S100A8 and S100A9 and lipid metabolism, aligning with findings of the current study. *FFAR4* encodes a protein belonging to the rhodopsin family of G-protein-coupled receptors, serving as a receptor for free fatty acids, including omega-3. Omega-3 has beneficial effects on obesity, type II diabetes mellitus, and other metabolic diseases. The activation of *FFAR4* by omega-3 suggests that high *FFAR4* expression in adipose tissue plays a crucial role in improving the nutritional composition of animal fat [43]. The observed differential expression of *FFAR4* in the samples we tested indicates variation in the omega-3 fatty acid content of different adipose tissues. In addition to *FFAR4* being associated with the synthesis of unsaturated fatty acids, *ALOX15* and *SCD* were also enriched in unsaturated fatty acid biosynthesis, indicating their involvement in unsaturated fatty acid biosynthesis in sheep adipose tissue. Further exploration of their regulatory effects can provide a theoretical basis for increasing the unsaturated fatty acid content of sheep adipose tissue.

It was interesting to discover that the novel genes *LOC101117229* and *LOC101122246596* were significantly enriched in pathways related to fat digestion and absorption, strongly indicating their close association with lipid metabolism. Additionally, the novel gene NC_056075.1.94 found alongside FFAR4 and INSIG1, was highly significantly enriched in the entries related to adipocyte differentiation. Glycerol levels were increased in FFAR4-lacking mice compared to littermate controls [44], and INSIG1 serves as a feedback mediator of cholesterol and fatty acid synthesis in animals [45]. This novel gene NC_056075.1.94 may play a pivotal role in the adipocyte differentiation process. This finding will serve as a reference for subsequent studies on the genetic regulation of adipocyte differentiation.

### 4.3. Enrichment and Roles of DEIs in Fat Metabolism

In this study, DEIs implicated in fat metabolism exhibited significant enrichment in processes such as fat deposition, lipid transport, and cholesterol synthesis. Notably, DEIs such as NC_056079.1.98.44, NC_056069.1.1013.11, and NC_056057.1.378.11 were concurrently enriched in fat digestion and absorption, the extracellular matrix (ECM) receptor interaction pathway, fatty acid biosynthesis, and fatty acid metabolism. The enrichment in various pathways, including lipid metabolism and the PPAR signaling pathway, suggested that these transcripts play crucial regulatory roles in fat metabolism.

The novel transcripts, NC_056066.1.1866.16 and NC_056066.1.1866.22, were significantly enriched in white fat cell differentiation, suggesting their involvement in adipocyte differentiation within the adipose tissue of 8-month-old sheep. Brown adipose tissue (BAT) plays a crucial role as a thermogenic tissue crucial for maintaining core body temperature [46]. White adipose tissue (WAT) is highly adaptable and can transform into BAT under the influence of factors such as physical activity and cold stimulation, a phenomenon known as the browning of WAT [47]. Activating BAT and inducing WAT browning can enhance glycolipid uptake and reduce the reliance on insulin secretion, representing a novel strategy to ameliorate disorders of glycolipid metabolism and insulin resistance in mammals [48].

Among the differentially expressed isoforms (DEIs) in the PPI networks of SF and VF, the hub isoforms, including NC_056055.1.1314.108, NC_056057.1.1402.33, and NC_056057.1.1402.7 (ranking among the top five), were all enriched in the PI3K-AKT KEGG pathway. Studies have indicated that hesperidin can alleviate hepatic steatosis by activating the liver Pl3k/AKT-Nrf2 pathway [49], and fluoxetine may regulate glucose and lipid metabolism in diabetic rats through the PI3K-AKT signaling pathway [50]. The PI3K-AKT-mTOR pathway plays a crucial role in the synthesis and secretion of triacylglycerol [51]. It is inferred that the newly identified transcripts in this study, particularly NC_056055.1.1314.108, NC_056057.1.1402.33, and NC_056057.1.1402.7, are key genes in sheep fat metabolism, and their mechanisms warrant further investigation.

Connexin, a protein forming gap junctions and non-antagonistic semi-channels of the cell membrane, plays a crucial role in fat formation and maintenance of adipose tissue homeostasis. At different stages of fat formation, the expression and function of connexin 43 (Cx43) are regulated differently. During the initial process of fat formation, connexins are highly phosphorylated. In the intermediate state of adipocyte differentiation, Cx43 is translocated from the membrane and phosphorylation is reduced by proteasome degradation [52]. In the DEI PPI networks of TF and SF, the hub isoforms, including XM_012180724.3, NC_056054.1.4780.11, NC_056064.1.197.5, NC_056064.1.1711.2, and isoforms XM_012180724.3, NC_056054.1.4780.11, NC_056064.1.1711.2, and Xm_015093599.3, were all enriched in the Proteasome KEGG pathway. This suggests that these five isoforms may be closely related to adipocyte differentiation, warranting attention to their mode of action.

In the PPI network of TFV and VF DEIs, the top five hub isoforms interacted with each other. The novel isoform NC_056055.1.1036.4 was enriched in the HIF-1 signaling pathway, insulin signaling pathway, and Pl3K-Akt signaling pathway. Genetic defects in HIF-1α have been associated with increased lipid accumulation, impairing the host protective anti-leishmanic function of myeloid cells [53]. Studies have shown that hypoxia-induced lipid accumulation in hepatocytes involves both HIF-1α and HIF-2α. This occurs through the reduction of PGC-1α-mediated fatty acid β-oxidation [54]. Therefore, it can be speculated that the novel isoform NC_056055.1.1036.4 is closely related to lipid accumulation.

This study has several limitations. Firstly, mixed-pool sequencing was adopted. Secondly, the analysis was conducted only at the transcriptome level. Thirdly, the analysis was confined to pre-transcription data. In future studies, post-transcription analysis will be conducted, and multi-omics data will be comprehensively utilized to validate the DEGs and DEIs.

## 5. Conclusions

Numerous DEGs and DEIs were identified in the subcutaneous, tail, and visceral adipose tissue of sheep. Key genes, such as *ACADL*, *ACSL6*, *COL1A1*, *COL1A2*, *FFAR4*, and *SCD*, exhibit differential expression in these adipose tissues and, therefore, regulate adipose metabolism. The novel gene *NC_056060.1.2536* is implicated in triglyceride metabolism, while the novel transcripts NC_056066.1.1866.16 and NC_056066.1.1866.22 are associated with white adipocyte differentiation. These findings contribute to a deeper understanding of the regulatory mechanisms underlying adipose tissue metabolism in sheep.

## Figures and Tables

**Table 1:** Comparison of FLNC mean PID values pre- and post-correction.

Feature	Number of FLNC Reads	Mean PID (%)	
		Pre-Correction	Post-Correction
only pre-correction mapped	9	51.86	–
only post-correction mapped	1075	–	86.79
pre PID > post PID	105,215	98.89	97.31
pre PID = post PID	372,471	99.38	99.38
pre PID < post PID	241,094	98.83	99.29
Total	719,864	98.97	99.03

FLNC, full-length non-chimeric; PID, percentage of identity.

**Table 2:** Classification of pre-correction, post-correction, and merged FLNC libraries after mapping to the reference genome.

Feature	Pre-Correction	Post-Correction	Merged
unmapped	1459 (0.20%)	393 (0.05%)	384 (0.05%)
multiple best mapped	5881 (0.82%)	6017 (0.84%)	5633 (0.78%)
low-PID sequences	17,250 (2.40%)	27,294 (3.79%)	14,551 (2.02%)
high-quality mapped	695,658 (96.59%)	686,544 (95.32%)	699,680 (97.14%)

FLNC, full-length non-chimeric; PID, percentage of identity.

**Table 3:** Comparison of genome annotation between the reference genome and PacBio sequencing.

Feature	Loci of Reference Annotation	Loci of PacBio Annotation
Loci	25,712	60,276
Loci < 1 K	5124 (19.93%)	11,652 (19.33%)
Loci 1–2 K	6032 (23.46%)	22,074 (36.62%)
Loci 2–3 K	4632 (18.01%)	14,657 (24.32%)
Loci ≥ 3 K	9924 (38.60%)	11,893 (19.73%)
Total	68,644	111,302

**Table 4:** The MCC values of the top five hub DEIs among different tissues in PPI network.

Group	Node_Name	MCC	Gene_Name
SF vs. VF	NC_056057.1.1402.7	67	*COL1A2*
	XM_004007726.5	67	*COL1A2*
	NC_056057.1.1402.33	59	*COL1A2*
	NC_056057.1.1402.18	57	*COL1A2*
	NC_056074.1.334.7	51	*EEF1G*
TF vs. SF	NC_056064.1.197.5	1.32 × 10^9^	*PSMD11*
	NC_056054.1.4780.11	1.32 × 10^9^	*PSMD4*
	XM_015093596.3	1.32 × 10^9^	*PSMD1*
	NC_056064.1.1711.2	1.32 × 10^9^	*PSMD3*
	XM_012180724.3	1.32 × 10^9^	*PSMC6*
TF vs. VF	NC_056067.1.612.3	8.72 × 10^13^	*RPS19*
	NC_056062.1.131.5	8.72 × 10^13^	*RPL8*
	NC_056077.1.54.1	8.72 × 10^13^	*RPS2*
	NC_056056.1.2665.21	8.72 × 10^13^	*RPL3*
	NC_056055.1.1036.4	8.72 × 10^13^	*RPS6*

SF, subcutaneous fat; TF, tail fat; VF, visceral fat. MCC, Mattews Correlation Coefficient.

## Data Availability

Data are contained within the article and supplementary materials.

## Supplementary Materials

The following supporting information can be downloaded at: https://www.mdpi.com/article/10.3390/ani14010007/s1, Figure S1: Overall description of full-length sequencing and error correction; Figure S2: KEGG and GO enrichment; Table S1: Primer synthesis sequence of target gene; Table S2: Length distribution of isoforms based on NCBI RefSeq (Annotation) and full-length sequencing conducted in the present study (PacBio); Table S3: Statistics for the functional annoation of novel isoforms using different databases; Table S4: Fusion genes; Table S5: Genes with more than five alternative polydenylation; Table S6: Statistics for the production of clean reads; Table S7: Mapping of clean reads to the reference genome; Table S8: Mapping of clean reads to the PacBio + RefSeq merged transcript library; Table S9: Statistics of the number of genes in different expression levels (FPKM); Table S10: Statistics of the number of isoforms in different expression levels (FPKM).
