# Supplementary material for: Full-Length Transcriptome and Gene Expression Analysis of Different Ovis aries Adipose Tissues Reveals Transcript Variants Involved in Lipid Biosynthesis
Source: Animals (Basel). 2023 Dec 19;14(1):7. doi: 10.3390/ani14010007 (PMC10777924; doi:10.3390/ani14010007)
Supplement: Supplementary file 1 [file animals-14-00007-s001.zip › animals-2699269-supplementary/Supplementary Figures.pdf]

Supplementary Figures

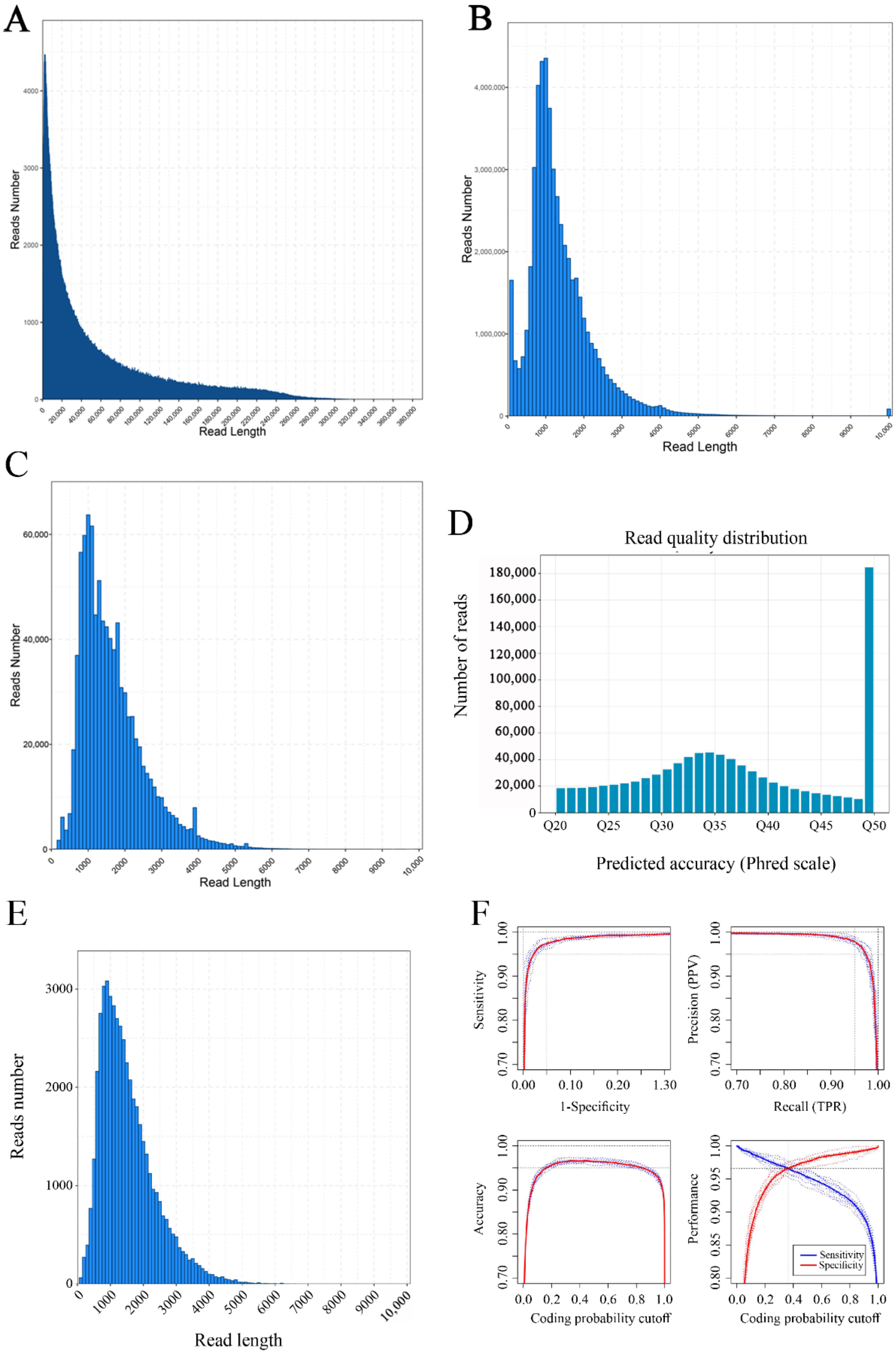

Figure S1: Overall description of full-length sequencing and error correction.

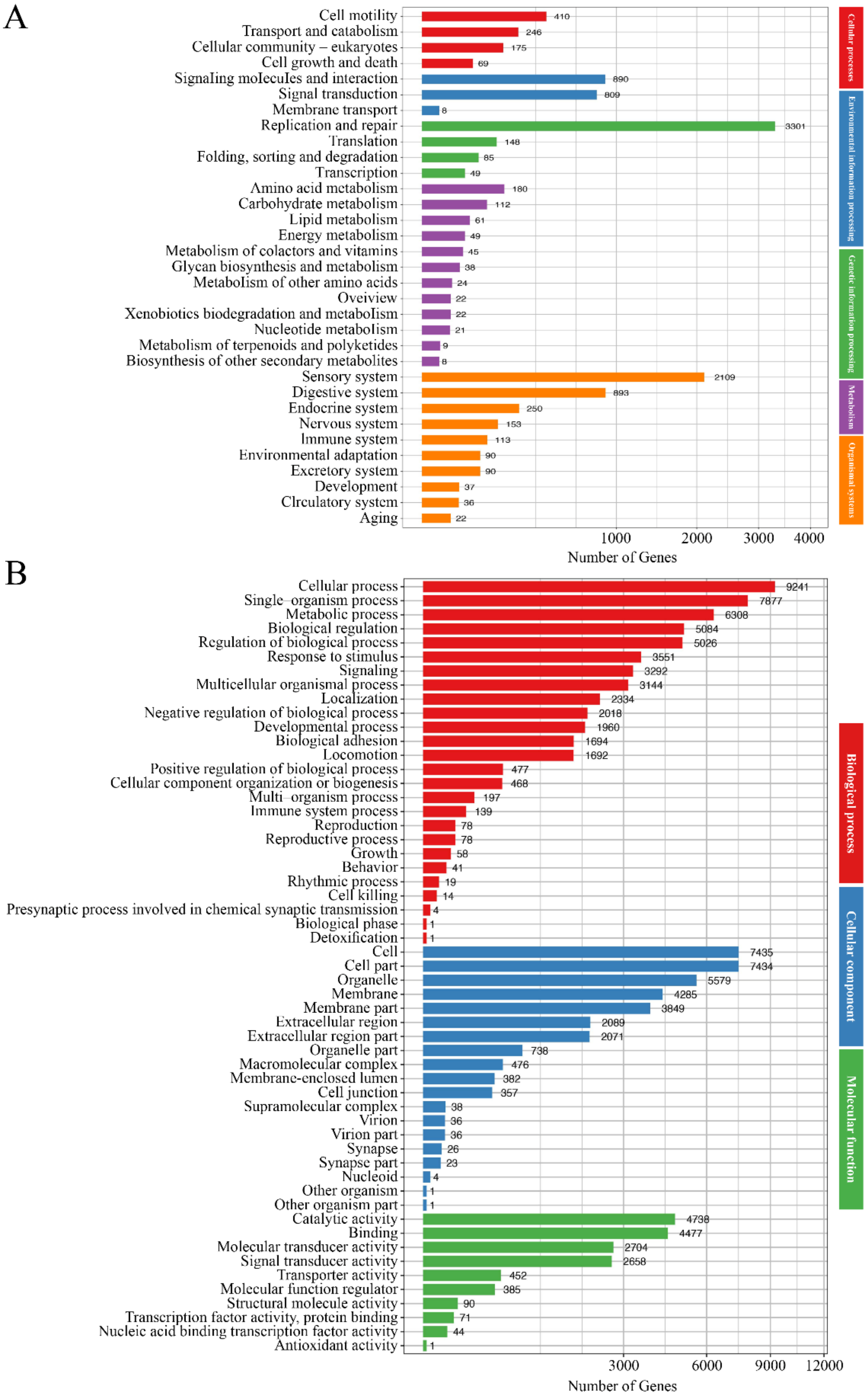

Figure S2: KEGG and GO enrichment.
